# A Congenic C57BL/6 *rd1* Mouse Model for Retinal Degeneration Research

**DOI:** 10.1016/j.exer.2025.110804

**Published:** 2025-12-09

**Authors:** Laurel C. Chandler, Apolonia Gardner, Constance L. Cepko

**Affiliations:** 1 Departments of Genetics and Ophthalmology, Blavatnik Institute, Harvard Medical School, Boston, MA 02115.; 2 Virology Program, Harvard Medical School, Boston, MA 02115.; 3 Howard Hughes Medical Institute, Chevy Chase, MD 20815.

**Keywords:** Pde6b, rods, retinal degeneration, retinitis pigmentosa, congenic, inbred, electroretinogram, optomotor, C57BL/6 with rd1 mutation, C57BL/6J.*Pde6b*^*rd1*^ strain

## Abstract

Retinitis pigmentosa is an inherited retinal disease caused by thousands of mutations in over 100 different genes. The most widely used mouse model for retinitis pigmentosa has the retinal degeneration 1 (*rd1*) mutation in the *Pde6b* gene, which elicits rapid retinal degeneration and vision loss. A major limitation of these models is that these *rd1* strains are not congenic, which prevents the use of appropriate controls. Furthermore, many strains have mutations in other genes which introduces genetic variability and may confound results. To address this issue, we backcrossed the *rd1* allele from FVB mice onto a C57BL/6J genetic background over many generations, producing a C57BL/6J.*Pde6b*^*rd1*^ strain that was confirmed to be congenic to C57BL/6J mice. We show that this strain recapitulates the electroretinogram and optomotor results expected for mouse strains containing the *rd1* mutation. Examination of retinal structure in cross sections of eyes isolated from C57BL/6J.*Pde6b*^*rd1*^ mice show a degree of thinning of the outer nuclear layer expected for a *rd1* mutation, resulting in nearly complete loss of the outer nuclear layer by postnatal day 35. We anticipate that this C57BL/6J.*Pde6b*^*rd1*^ strain could become an asset for the field of retinitis pigmentosa research.

Retinitis pigmentosa (RP) is the most common cause of inherited retinal degeneration, affecting ~2 million people globally.^1,2^ The disease manifests as an initial loss of rods, affecting night vision, followed by the gradual degeneration of cones, which affects daylight vision.^3^ This disease can be caused by a wide variety of mutations in any of >100 genes linked to RP (RetNet, https://RetNet.org/).^4^ The PDE6B gene, encoding for the phosphodiesterase 6 beta subunit, plays a crucial role in the light-responsive signal transduction pathway in rod photoreceptors.^5^ To model RP in mice, there are several strains with mutations in *Pde6b*, including those which carry the retinal degeneration 1 (*rd1*) allele, which leads to rapid retinal degeneration and vision loss. The *rd1* mutation includes an intronic murine leukemia viral insertion in exon 1 and a nonsense point mutation in exon 7, which together lead to complete loss of PDE6β protein.^6,7^ As the first and best-studied retinal degeneration mouse model, mice exhibiting the *rd1* mutation are widely used in RP research, with >1,000 articles published using this allele (PubMed search term “rd1”).^8^ However, strains which contain this mutation, including FVB, C3H, CBA, and SJL, have mutations in other genes, which may confound analyses.^9^ It has also been difficult to include the appropriate control strain in these studies as there is no congenic *rd1* mouse model, unlike other RP mouse strains such as *rd10* and P23H which do have congenic controls.^10^ In order to generate a congenic *rd1* mouse, we backcrossed FVB mice (strain #207, Charles River Laboratories), which carry the *rd1* mutation, with C57BL/6J mice (strain #000664, The Jackson Laboratory) for many generations until the *rd1* allele was present on a C57BL/6J background. These C57BL/6J.*Pde6b*^*rd1*^ mice were assessed using Jackson Laboratory’s specific SNP Genome Scanning service and demonstrated 100% background identity to C57BL/6J mice and 0% identity to the FVB strain (Table 1). This study was approved by the Institutional Animal Care and Use Committee of Harvard University.

To characterize visual function in the C57BL/6J.*Pde6b*^*rd1*^ mice, electroretinogram (ERG) analysis was performed. To measure rod and cone function, scotopic and photopic ERGs were measured, respectively, using an Espion E3 System (Diagnosys LLC) as previously described.^11^ All animals were dark adapted for at least four hours prior to recording. Mice were anesthetized by intraperitoneal injection of ketamine and xylazine (100 and 10 mg/kg, respectively). Tropicamide 1% eye solution (Bausch + Lomb) was used to dilate the pupil. Dim flashes were applied to elicit a scotopic ERG response at a 0.1 candela (cd) s/m^2^ intensity. The scotopic b-wave response was assessed in both wildtype C57BL/6J and C57BL/6J.*Pde6b*^*rd1*^ mice at postnatal day 20 (P20) in low light conditions to isolate the rod photoreceptors and their downstream connection to ON-bipolar cells. There was a statistically significant loss of scotopic ERG response in C57BL/6J.*Pde6b*^*rd1*^ compared to C57BL/6J mice (Figure 1A). All animals were then light adapted for six minutes with a white light background of 30 cd s/m^2^. Multiple white flashes were applied to elicit photopic ERG responses at 1 (peak), 10 (peak), 100 (xenon), and 1000 (xenon) cd s/m^2^ intensities with a background light of 30 cd s/m^2^. At all intensities, the photopic b-wave, which primarily measures the response from cone ON-bipolar cells, was significantly reduced in C57BL/6J.*Pde6b*^*rd1*^ compared to C57BL/6J mice (Figure 1B and C). There was no further reduction in the b-wave amplitude when assessing the ERG response at 10 cd s/m^2^ at P30 and 40, suggesting that most of the response had already been lost by P20 (Figure 1D).

Although the ERG response is rapidly lost in RP mouse models, the optomotor response can be retained for significantly longer.^11^ The optomotor response was measured using the OptoMotry System (CerebralMechanics) under photopic conditions with a background luminance of ~70 cd s/m^2^ as previously described.^12^ The contrast of the black and white stripes were displayed at 100% and the spatial frequency was set to 1.5 Hz. To assess visual acuity, the program changed the frequency of the stripes (cycles/degree) and the direction of the rotation (clockwise vs counterclockwise), while the examiner recorded whether head tracking was present. This was continued until the program had reached a threshold of acuity for each animal. Visual acuity was assessed every five days from P25–45 in C57BL/6J and C57BL/6J.*Pde6b*^*rd1*^ mice. At P25, there was no significant difference between the visual acuity of the C57BL/6J and C57BL/6J.*Pde6b*^*rd1*^ mice, however from P30 onwards there was a gradual loss of vision in the C57BL/6J.*Pde6b*^*rd1*^ mice while wildtype mice remained unchanged (Figure 1E).

To visualize retinal morphology in the C57BL/6J.*Pde6b*^*rd1*^ mice compared to FVB and C57BL/6J controls, eyes were harvested from mice and prepared for sectioning as previously described.^13^ Sections were cut at a thickness of 20 μm on a CM 3050S cryostat (Leica Microsystems) prior to staining with DAPI. Samples were mounted with Fluoromount G (Southern Biotech) under coverslips and imaged on an Olympus VS200 Slide Scanner with a UPlan X Apo 10x/0.4 Air objective. Representative eyecup sections at P15 and P35 for each of the three mouse strains showed the outer nuclear layer (ONL) to be comparable in the C57BL/6J.*Pde6b*^*rd1*^ and FVB mouse strains, both of which were significantly thinner than the wildtype C57BL/6J controls (Figure 1F) suggesting substantial retinal degeneration by this age. The thickness of the ONL was measured at 500, 750, and 1000 μm intervals both dorsal and ventral of the optic nerve. There was a significant reduction in ONL thickness in both FVB and C57BL/6J.*Pde6b*^*rd1*^ mice at P15 relative to C57BL/6J controls (Figure 1G). This thickness was further reduced to ~1 layer of nuclei in the FVB and C57BL/6J.*Pde6b*^*rd1*^ mice at age P35, whereas C57BL/6J controls retained healthy ONL thicknesses (Figure 1H).

In this study, we generated a congenic mouse line carrying the *Pde6b*^*rd1*^ allele. This allele was transferred onto the C57BL/6J background in-house by backcrossing the FVB *rd1* mutation onto the C57BL/6J mouse background. After confirming that this new C57BL/6J.*Pde6b*^*rd1*^ strain has the background of the C57BL/6J strain, with no detectable alleles from the FVB strain, we performed both functional and structural assays to determine the extent and kinetics of degeneration in this mouse line. ERG analyses showed a significant reduction in scotopic (rod-mediated) and photopic (cone-mediated) vision by P20 that didn’t undergo further reduction at later ages, indicating that the C57BL/6J.*Pde6b*^*rd1*^ strain had lost most visual function by weaning age. We also performed an optomotor assay to assess visual acuity from P25-P45, which showed a steady decline in the C57BL/6J.*Pde6b*^*rd1*^ strain until a near total loss was achieved by P45. Consistent with the loss of visual function, ONL thickness was markedly reduced in the C57BL/6J.*Pde6b*^*rd1*^ strain relative to wildtype C57BL/6J controls at P15. By P35, the ONL was reduced to ~1 nuclei layer in the C57BL/6.*Pde6b*^*rd1*^strain. ONL thickness was reduced to approximately the same extent in FVB and C57BL/6.*Pde6b*^*rd1*^ at both timepoints demonstrating that retention of the *rd1* allele from the FVB strain is sufficient for retinal degeneration.

There are other congenic/coisogenic strains of *Pde6b* mutant mice now available, including those generated by Chirinskaite and colleagues using CRISPR-Cas9 KO technology^14^ and another by The Jackson Laboratory generated via a chemically induced mutation (strain #004766). However, sequencing demonstrated that these other *Pde6b* mutant strains contain disruptions in addition to the *rd1* mutation and have slower kinetics and extents of degeneration than *rd1* mutant mice.^14^ The C57BL/6J.*Pde6b*^*rd1*^ strain described in this study optimally combines the benefits of a congenic mouse model with rapid-onset retinal degeneration.

## Figures and Tables

**Figure 1.**
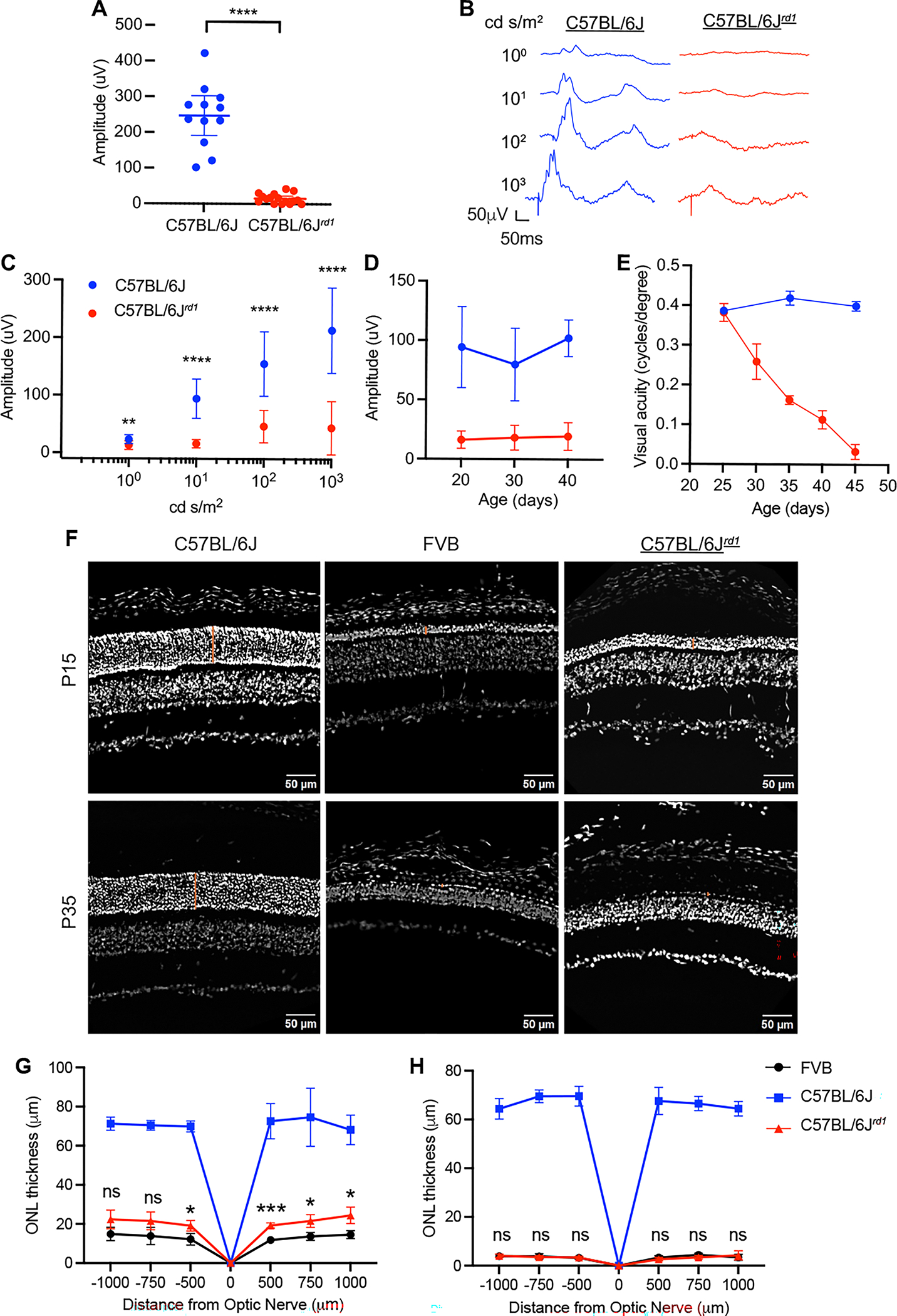
Examination of the visual function, visual acuity, and outer nuclear layer (ONL) thickness in C57BL/6J mice with the *rd1* mutation. (A) Dark-adapted scotopic response at a 0.1 cd s/m^2^ flash intensity in P20 C57BL/6J (n=12) and C57BL/6J.*Pde6b*^*rd1*^ (C57BL/6J^*rd1*^) (n=16) mice. ****p<0.0001, unpaired T-test. (B) Representative light-adapted electroretinogram (ERG) traces in P20 C57BL/6J or C57BL/6J^rd1^ mice subject to a range of flash intensities. (C) Light-adapted photopic b-wave in P20 mice at a range of flash intensities (n=12). **p=0.0073, ****p<0.0001, two-way ANOVA with Šídák’s multiple comparison test. (D) Photopic b-wave at a flash intensity of 100 cd s/m^2^ over time at ages P20, 30 and 40 (n=12). (E) Visual acuity of wildtype C57BL/6J compared to C57BL/6J^*rd1*^ mice as measured by the number of cycles/degree using the optomotor assay (n=6–10 per group). (F) Representative retinal sections from wildtype C57BL/6J, FVB and C57BL/6J^*rd1*^ mice at P15 and P35. Sections are stained with DAPI (white). Orange line denotes the ONL. Scale bar is 50 microns. ONL thickness in (G) P15 and (H) P35 mice measured using the DAPI channel at intervals along the dorsal-ventral axis with the most ventral point at −1000 μm and most dorsal point at 1000 μm from the optic nerve. Images were analyzed using OlyVIA software (n=3–5 per group). *p=0.0209 at −500 μm, ***p=0.0002 at 500 μm, *p=0.0210 at 750 μm, *p=0.0265 at 1000 μm from the optic nerve, two-way ANOVA with Tukey’s multiple comparison test. Significance values only shown for multiple comparisons between FVB and C57BL/6J^*rd1*^, comparisons of both degenerate strains with C57BL/6J are all statistically significant.

**Table 1.** Determination of the background identity of the C57BL/6.*Pde6b*^*rd1*^ strain.

Sample	FVB/NJ (% identity)	C57BL/6J (% identity)
C57BL/6J.*Pde6b^rd1^* #1	0.00	100.00
C57BL/6J.*Pde6b^rd1^* #2	0.00	100.00
C57BL/6J.*Pde6b^rd1^* #3	0.00	100.00
C57BL/6J.*Pde6b^rd1^*#4	0.00	100.00
C57BL/6J.*Pde6b^rd1^* #5	0.00	100.00
C57BL/6J control	0.00	100.00
FVB/NJ control	100.00	0.00
C57BL/6J × FVB/NJ heterozygous	50.00	50.00

Table 1: **Genotypes of mice from crosses of FVB to C57BL/6J mice.** FVB mice with the *rd1* allele were backcrossed to wildtype C57BL/6J until SNP genotyping, carried out by the Jackson Laboratory’s SNP Genome Scanning service, revealed that the *rd1* allele was on a C57BL/6J background. The results from five individual mice from these backcrosses, as well as parental and F1 mice, are shown.

## References

[1] Suleman N Current understanding on Retinitis Pigmentosa: a literature review. Front Ophthalmol (Lausanne) 5, 1600283 (2025). 40575766 10.3389/fopht.2025.1600283 PMC12198980

[2] Hamel C Retinitis pigmentosa. Orphanet J Rare Dis 1, 40 (2006). 17032466 10.1186/1750-1172-1-40 PMC1621055

[3] Kamde SP & Anjankar A Retinitis Pigmentosa: Pathogenesis, Diagnostic Findings, and Treatment. Cureus 15, e48006 (2023). 38034182 10.7759/cureus.48006 PMC10686897

[4] RetNet. https://RetNet.org/.

[5] Kuehlewein L Clinical phenotype of PDE6B-associated retinitis pigmentosa. Int. J. Mol. Sci. 22, 2374 (2021). 33673512 10.3390/ijms22052374 PMC7956818

[6] Bowes C Localization of a retroviral element within the rd gene coding for the beta subunit of cGMP phosphodiesterase. Proc Natl Acad Sci U S A 90, 2955–2959 (1993). 8385352 10.1073/pnas.90.7.2955 PMC46215

[7] Pittler SJ & Baehr W Identification of a nonsense mutation in the rod photoreceptor cGMP phosphodiesterase beta-subunit gene of the rd mouse. Proc Natl Acad Sci U S A 88, 8322–8326 (1991). 1656438 10.1073/pnas.88.19.8322 PMC52500

[8] Kalloniatis M, Nivison-Smith L, Chua J, Acosta ML & Fletcher EL Using the rd1 mouse to understand functional and anatomical retinal remodelling and treatment implications in retinitis pigmentosa: A review. Exp Eye Res 150, 106–121 (2016). 26521764 10.1016/j.exer.2015.10.019

[9] van Wyk M, Schneider S & Kleinlogel S Variable phenotypic expressivity in inbred retinal degeneration mouse lines: A comparative study of C3H/HeOu and FVB/N rd1 mice. Mol Vis 21, 811–827 (2015). 26283863 PMC4522243

[10] Sakami S Probing mechanisms of photoreceptor degeneration in a new mouse model of the common form of autosomal dominant retinitis pigmentosa due to P23H opsin mutations. J Biol Chem 286, 10551–10567 (2011). 21224384 10.1074/jbc.M110.209759 PMC3060508

[11] Xue Y AAV-Txnip prolongs cone survival and vision in mouse models of retinitis pigmentosa. Elife 10, (2021). 10.7554/eLife.66240 PMC8081528 33847261

[12] Xiong W AAV cis-regulatory sequences are correlated with ocular toxicity. Proc. Natl. Acad. Sci. U. S. A. 116, 5785–5794 (2019). 30833387 10.1073/pnas.1821000116 PMC6431174

[13] Chandler LC, Gardner A & Cepko CL RPE-specific MCT2 expression promotes cone survival in models of retinitis pigmentosa. Proc Natl Acad Sci U S A 122, e2421978122 (2025). 40178895 10.1073/pnas.2421978122 PMC12002273

[14] Chirinskaite AV Does Background Matter? A Comparative Characterization of Mouse Models of Autosomal Retinitis Pigmentosa rd1 and Pde6b-KO. Int J Mol Sci 24, (2023). 10.3390/ijms242417180 PMC10742838 38139011

